# TIME-VARYING ASSOCIATIONS BETWEEN SOCIAL INTERACTION MODALITY AND POSITIVE AFFECT DURING TIMES OF CHALLENGE

**DOI:** 10.1093/geroni/igac059.2173

**Published:** 2022-12-20

**Authors:** Tiana Broen, Yoonseok Choi, Theresa Pauly, Elizabeth Zambrano Garza, Denis Gerstorf, Christiane Hoppmann

**Affiliations:** University of British Columbia, Vancouver, British Columbia, Canada; University of British Columbia, Vancouver, British Columbia, Canada; University of Zurich, Zurich, Zurich, Switzerland; University of British Columbia, Vancouver, British Columbia, Canada; Humboldt Universität zu Berlin, Berlin, Berlin, Germany; University of British Columbia, Vancouver, British Columbia, Canada

## Abstract

The COVID-19 pandemic compromised psychological wellbeing, especially given public health guidance that restricted social contact. Engaging in daily social interactions is especially important for maintaining wellbeing, and virtual interactions may be a surrogate for face-to-face interactions when in-person contact is not possible. Embracing the idea that virtual communication may facilitate social connectedness, this project seeks to better understand the potential of virtual social interactions for supporting wellbeing. Using repeated-daily-life-assessments from an adult lifespan sample (N=164, Mage=42.53 years), we examined how number of virtual and face-to-face social interactions were associated with positive affect in individuals’ daily lives. We expected that face-to-face interactions would have a stronger relationship with positive affect than virtual social interactions, but that engaging in virtual interactions would be positively related to affect during these unprecedented times. In line with our hypotheses, number of daily in-person (r(812)=0.14, p<.001) and total number of social interactions (r(812)=0.14, p<.001) were associated with higher positive affect at the bivariate level; number of daily virtual social interactions showed a positive trend with affect (r(812)=0.06, p=.07). Multilevel models including quantity of face-to-face and virtual interactions indicate that only face-to-face interactions were associated with concurrent elevations in positive affect (b=0.58, p=.03) and overall higher positive affect (b=0.72, p<.001). Future analyses will examine whether social interactions involving close ties are more powerful drivers of positive affect than distal ties, whether everyday closeness to others mediates the relationship between social interaction modality and positive affect, and we will unpack age as a moderator in these relationships.

